# Shikonin and Juglone Inhibit *Mycobacterium tuberculosis* Low-Molecular-Weight Protein Tyrosine Phosphatase a (Mt-PTPa)

**DOI:** 10.3390/biotech12030059

**Published:** 2023-09-20

**Authors:** Abdulhakeem O. Sulyman, Jessie Fulcher, Samuel Crossley, Amos A. Fatokun, Femi J. Olorunniji

**Affiliations:** 1 Department of Biochemistry, Faculty of Pure and Applied Sciences, Kwara State University, Malete 241103, Nigeria; 2 School of Pharmacy & Biomolecular Sciences, Faculty of Science, Liverpool John Moores University, Byrom Street, Liverpool L3 3AF, UK

**Keywords:** mycobacterium, protein tyrosine phosphatase, inhibition, naphthoquinones, shikonin, juglone

## Abstract

Low-molecular-weight protein tyrosine phosphatases (LMW-PTPs) are involved in promoting the intracellular survival of *Mycobacterium tuberculosis* (Mtb), the causative organism of tuberculosis. These PTPs directly alter host signalling pathways to evade the hostile environment of macrophages and avoid host clearance. Among these, protein tyrosine phosphatase A (Mt-PTPa) is implicated in phagosome acidification failure, thereby inhibiting phagosome maturation to promote *Mycobacterium tuberculosis* (*Mtb*) survival. In this study, we explored Mt-PTPa as a potential drug target for treating *Mtb*. We started by screening a library of 502 pure natural compounds against the activities of Mt-PTPa in vitro, with a threshold of 50% inhibition of activity via a <500 µM concentration of the candidate drugs. The initial screen identified epigallocatechin, myricetin, rosmarinic acid, and shikonin as hits. Among these, the naphthoquinone, shikonin (5, 8-dihydroxy-2-[(1R)-1-hydroxy-4-methyl-3-pentenyl]-1,4-naphthoquinone), showed the strongest inhibition (IC_50_ 33 µM). Further tests showed that juglone (5-hydroxy-1,4-naphthalenedione), another naphthoquinone, displayed similar potent inhibition of Mt-PTPa to shikonin. Kinetic analysis of the inhibition patterns suggests a non-competitive inhibition mechanism for both compounds, with inhibitor constants (Ki) of 8.5 µM and 12.5 µM for shikonin and juglone, respectively. Our findings are consistent with earlier studies suggesting that Mt-PTPa is susceptible to specific allosteric modulation via a non-competitive or mixed inhibition mechanism.

## 1. Introduction

Tuberculosis (TB), a communicable disease caused by Mycobacterium tuberculosis (Mtb) that is transmitted through the air, continues to be a major global health concern, having resulted in a staggering 1.5 million fatalities worldwide in the year 2020, and it is estimated to infect one-quarter of the world’s population, with approximately 10 million people falling ill with TB in 2020 [1]. Currently, drug-susceptible TB can be successfully treated with a six-to-nine-month course of antimicrobial drugs. However, with increasing HIV co-infection and the accelerated ability of microbes to develop resistance to multiple antibiotics, the incidence of TB remains a worldwide health threat. The rise of multidrug-resistant tuberculosis (MDR-TB) and extensively drug-resistant tuberculosis (XDR-TB) represents a growing threat to public health. This has highlighted the need for the discovery of antibacterial agents with novel mechanisms of action [2]. In fact, in the last two decades, only three novel anti-TB drugs have been approved to treat MDR-TB [3]. Thus, there is an urgent need to identify new target entities for the long-term goal of providing potential candidates for the development of novel anti-TB drugs.

As the demand for novel antimicrobial treatments is inescapable, and since many of the first antibiotics utilised natural products, efforts have focused on naturally occurring compounds in the hope of identifying novel therapeutic agents. An effective strategy proposed by many researchers for the discovery of novel antibiotics is to identify and target virulence factors responsible for bacterial survival in hosts [4].

Macrophages serve as the first line of immune defence against external pathogens, including *Mtb*. Therefore, the survival of bacteria within a living organism relies on the crucial interaction between host macrophages and invading microorganisms in vivo. Following inhalation, alveolar macrophages are responsible for the phagocytosis and translocation of *Mtb* into phagosomes. Upon interacting with the endocytic pathway, the phagosome membrane undergoes changes that allow for the recruitment of the hydrolases [5] and vacuolar H+ATPase (V-ATPase), a protein complex that performs as a proton pump [6]. The presence of V-ATPase causes the phagosome lumen to become acidic, indicating phagosome maturation [7], and the acid-activated hydrolases then proceed to precipitate the destruction of the invading pathogen.

After being phagocytosed, *Mtb* deploys an army of factors to avoid macrophage defences to enable its survival within the host. Among the factors secreted by *Mtb*, two low-molecular-weight protein tyrosine phosphatases (LMW-PTPs), namely mPTPa and mPTPb, represent promising therapeutic targets. These PTPs directly alter host signalling pathways to evade the hostile environment of macrophages and avoid host clearance [8], thereby promoting the intracellular survival of *Mtb*. Mt-PTPa blocks phagolysosome fusion [9], thus causing phagosome acidification failure and the arrest of phagosome maturation [10], while mPTPB is reported to circumvent immune clearance by disrupting host signalling pathways and suppressing macrophage-induced apoptosis [11].

Due to its more critical role in *Mtb*, investigators have focussed more on Mt-PTPb than Mt-PTPa in the search for potential inhibitors. Yet, the evidence shows that Mt-PTPa can be a target for the development of novel drugs for tuberculosis [12,13]. In this study, we focused on Mt-PTPa as a potential drug target for treating *Mtb*. The enzyme is implicated in phagosome acidification failure, thereby inhibiting phagosome maturation to promote *Mtb* survival [10]. It is known that THP-1 macrophages with Mt-PTPa deleted from *Mtb* show increased phagosome–lysosome fusion compared with macrophages containing Mt-PTPa [14].

Developing clinically favourable PTP inhibitors is complicated by selectivity issues. Many PTPs share a common dephosphorylation mechanism with the same conserved [C(X)_5_R(S/T)] active site sequence, and so, inhibitors against PTPs tend to fall victim to non-specific inhibition [10]. For PTP inhibitors to be regarded as clinically useful, they must be highly selective to avoid unwanted interaction with human or other mammalian LMW-PTPs, which may share a high degree of structural similarity with the intended target. Cell permeability is an additional factor that has hindered the development of therapeutic PTP inhibitors [15]. As the phosphotyrosine substrates for PTPs are anionic, prospective inhibitors are often also highly negatively charged to mimic the substrate and conform with the PTP’s positively charged active site. Despite the abundance of potential *Mtb* inhibitors described in the literature, this anionic nature renders many compounds cell-impermeable and unable to reach the target enzyme [10]. Thus, the continued development and investigation of inhibitors that exhibit high affinity, but are less negatively charged, is necessary for improving the development of Mt-PTPa and Mt-PTPb inhibitors.

In this report, we show that Mt-PTPa is inhibited in vitro by two naphthoquinones, shikonin (5, 8-dihydroxy-2-[(1R)-1-hydroxy-4-methyl-3-pentenyl]-1,4-naphthoquinone) and juglone (5-hydroxy-1,4-naphthalenedione), in an allosteric manner, opening a new avenue for the potential development of novel treatments for tuberculosis.

## 2. Materials and Methods

### 2.1. Materials

All reagents and the substrate (p-nitrophenylphosphate) were purchased from Sigma-Aldrich, UK, and were of analytical grade. Further experiments using epigallochetin, myricetin, rosmarinic acid, shikonin, and juglone were conducted using samples obtained from Sigma-Aldrich, Poole, UK.

### 2.2. Compound Library Screening

The initial screen of 502 compounds was conducted using Screen-Well^®^ Natural Product Library (BML-2865 Version 7.1) in which each library compound was supplied as a 2 mg/mL solution in DMSO (Enzo Life Sciences; https://www.enzolifesciences.com/BML-2865/screen-well-natural-product-library/. Accessed on 22 September 2022). The library includes terpenoids, peptolides, flavones, coumarins, alkaloids, macrolides, isoflavones, and synthetic derivatives. Each compound was originally supplied at 2 mg/mL in DMSO and was diluted to a final concentration of 20 µg/mL to test their inhibition of Mt-PTPa in the initial library screening.

### 2.3. Cloning, Expression, and Purification of Mycobacterium tuberculosis PTPa

The molecular cloning protocols used in this study were based on the methods described by Sambrook and Russell [16] with some modifications. The codon-optimised sequences of Mt-PTPa, based on the published amino acid sequence (GenBank: AJW49631.1), were obtained from GeneArt (Invitrogen), Waltham, MA, USA. To construct the overexpression plasmid of Mt-PTPa (FEM87), the coding sequence was cloned into the pET-28a(+) vector (Novagen) between the NdeI and XhoI sites. The resulting plasmid contained an N-terminal hexahistidine tag (MGSSHHHHHHSSGLVPRGSHM followed by Mt-PTPa amino acid 2) to allow purification via nickel affinity chromatography. The correctness of the phosphatase open reading frame (ORF) in the expression plasmid was confirmed via sequencing.

The expression plasmid (pFEM87) was transformed into the BL21(DE3)pLysS strain, and positive transformants were selected using kanamycin (50 μg/mL). A starter culture of L-broth (5 mL) containing kanamycin (50 μg/mL) and chloramphenicol (25 μg/mL) was inoculated from a single transformant colony and grown overnight at 37 °C. Subsequently, 4 mL of the starter culture was added to 400 mL of L-broth containing kanamycin and chloramphenicol in 2-litre conical flasks. The cultures were grown at 37 °C with shaking at 250 rpm until the mid-log phase was reached (OD_600_ of 0.5–0.6).

To induce protein overexpression, the cultures were rapidly cooled down to 22 °C, and isopropyl-β-D-thiogalactopyranoside (IPTG) was added to a final concentration of 0.75 mM. The cultures were further grown for 18 h at 22 °C with shaking at 200 rpm. To harvest the cells, the culture was centrifuged at 4 °C, and the pellet was washed with a 100 mL buffer containing 20 mM Tris-HCl (pH 7.5) and 10 mM MgCl2. The washed pellet was collected via centrifugation and stored at −20 °C until further use.

The washed pellet was resuspended in 10 mL of a lysis buffer composed of 20 mM sodium phosphate (pH 7.4), 0.5 M NaCl, 1 mM dithiothreitol (DTT), 50 mM imidazole, and 1.2 mM phenylmethylsulfonyl fluoride (PMSF). PMSF was added to minimise post-lysis protease activity in the crude lysate. The cells were then sonicated at 4 °C (Vibra-Cell instrument, Sonics and Materials Inc.; 40% amplitude; 3 × 20 s bursts). After sonication, the cell suspension was centrifuged for 20 min at 4 °C, 48,000× *g*. The supernatant containing the soluble protein was subjected to metal affinity chromatography using a 1 mL HisTrap FF pre-packed column (GE Healthcare). The column was equilibrated with 10 mL of Buffer A (20 mM sodium phosphate, pH 7.4, 0.5 M NaCl, 1 mM DTT, and 50 mM imidazole) at a flow rate of 1 mL/minute before loading the protein-containing supernatant in Buffer A.

The column was then washed with 20 mL of Buffer A to remove unbound proteins and other cellular components present in the lysate. The target protein (Mt-PTPa) was eluted from the column using a linear gradient elution program with Buffer B (Buffer A supplemented with 500 mM imidazole) over 25 min (0–100% linear gradient). Fractions containing the target protein were collected, and their activity was immediately tested using the hydrolysis of p-nitrophenylphosphate. The purity of active fractions was determined via SDS-polyacrylamide gel electrophoresis. Selected fractions were dialyzed against 500 mL of Buffer C (25 mM Tris-HCl, pH 7.5, 1 mM DTT, 0.5 M NaCl, and 25% glycerol) for 16 h and stored at −20 °C. The protein concentration was determined using the absorbance at 280 nm (A280) and the molar extinction coefficient of Mt-PTPa, calculated using the online tool EMBOSS Pepstats (https://www.ebi.ac.uk/Tools/seqstats/emboss_pepstats/, accessed on 22 September 2022).

### 2.4. Determination of Phosphatase Activity and Inhibition Assays

Typically, phosphatase activity was determined following a protocol modified from Igunnu et al. [17]. Essentially, 10 μM of Mt-PTPa was added to the reaction mixture (100 µL) containing 50 mM Tris-HCl, pH 7.5, 50 mM NaCl, 0.1 mM EDTA, and 1 mM *p*-nitrophenyl phosphate as substrate. The concentrations of the substrate and any inhibitor being tested varied with the nature of the experiment, as detailed in the Results section. Reactions were typically started by the addition of the substrate and allowed to proceed for 10 min, after which, they were terminated by the addition of (100 µL) ‘Reaction Stop Buffer’ containing 25 mM Tris-HCl, pH 7.5, 25 mM EDTA, and 0.1% SDS. Absorbance change, A_405_, (characteristic for the reaction product p-nitrophenol) was measured using a CLARIOstar Plate Reader (BMG LABTECH). Activities are expressed as change in absorbance at 405 nm per minute.

In experiments in which the effects of inhibitors were tested, the enzyme was pre-incubated with the test compound in a buffered solution for 10 min, after which, the reaction was started by the addition of the substrate. Further details of the inhibition experiments are provided in the Results section.

## 3. Results

### 3.1. Initial Screening of Library of Chemical Compounds

We started by screening 502 pure natural compounds, derived from the Screen-Well^®^ Natural Product Library (Enzo Life Sciences; https://www.enzolifesciences.com/BML-2865/screen-well-natural-product-library/, accessed on 22 September 2022), against Mt-PTPa activity. Each compound was initially tested at a final concentration of 20 µg/mL. The compounds were dissolved in 100% dimethylsulfoxide (DMSO). For the inhibition assay, an 8 µL solution of each compound was included in a final reaction volume of 100 µL. For the control reactions, 8 µL of 100% DMSO was used in place of the compound solutions, giving a final concentration of 8% DMSO. The activity observed in the presence of 8% DMSO was normalised and taken as the uninhibited reaction. After the initial screening, we determined the molar concentrations of the 20 µg/mL solution of each compound and set a threshold of 50% inhibition of activity by a <500 µM concentration of the candidate compounds. From this initial screen, we identified four compounds: epigallocatechin, myricetin, rosmarinic acid, and shikonin.

### 3.2. Inhibition of Mt-PTPa by Epigallocatechin, Myricetin, Rosmarinic Acid, and Shikonin

**Figure 1:**
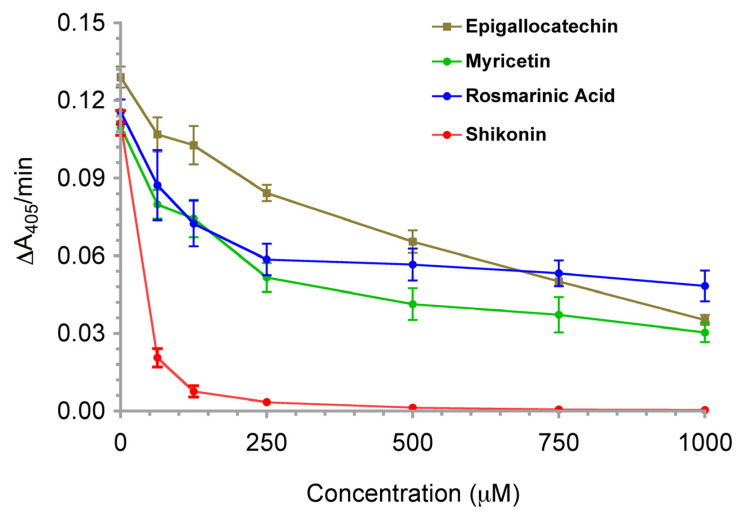
In vitro inhibition of Mt-PTPa via epigallocatechin, myricetin, rosmarinic acid, and shikonin. The hydrolysis of pNPP (100 µM) via Mt-PTPa (10 µM) in the presence of different concentrations of each compound (0, 63, 125, 250, 500, 750, and 1000 µM) was investigated in a total reaction volume of 100 µL. Reactions were incubated at 37 °C for 10 min, after which, they were terminated by the addition of 100 µL of ‘Reaction Stop Buffer’ (25 mM Tris-HCl, pH 7.5, 25 mM EDTA, and 0.1% SDS). Values are shown as mean ± SD (*n* = 3).

To determine the effectiveness of each of the four compounds identified from the initial screen, we investigated the effect of increasing concentrations of the compounds on the activity of Mt-PTPa. Figure 1 shows the concentration-dependent inhibition of Mt-PTPa (10 µM) by the four compounds we identified from the initial screen. Assays were carried out using a limiting concentration of pNPP (100 µM), and the effect of each compound was studied at 0, 63, 125, 250, 500, 750, and 1000 µM. The estimated IC_50_ values are shown in Table 1. Among these, shikonin showed strong inhibition (IC_50_ 33 µM) at a concentration that is potentially clinically relevant. This is consistent with reports in the literature of the inhibition of other protein tyrosine phosphatases via shikonin. Hence, we chose to focus our investigation on the mode of inhibition of PTP via shikonin in this work.

### 3.3. Shikonin and Juglone Are Potent Inhibitors of Mycobacterium Mt-PTPa

**Figure 2:**
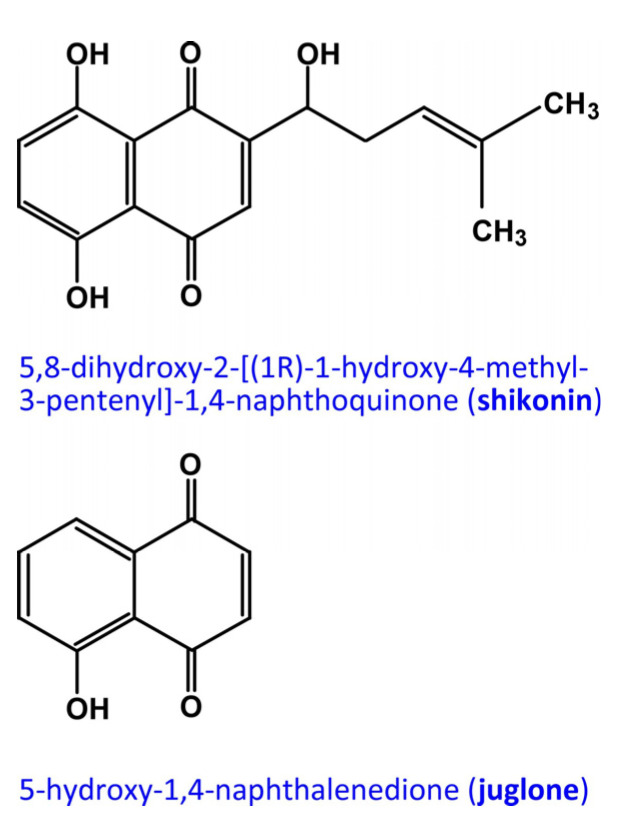
Chemical structures of shikonin and juglone.

Table 1 is consistent with the reported inhibition of other PTPs via shikonin and related naphthoquinones. To test if the inhibition of mPTPa is a common feature of naphthoquinones, we asked if juglone (5-hydroxy-1,4-naphthalenedione), another naphthoquinone with close structural similarity to shikonin (Figure 2), will have the same effect on Mt-PTPa.

**Figure 3:**
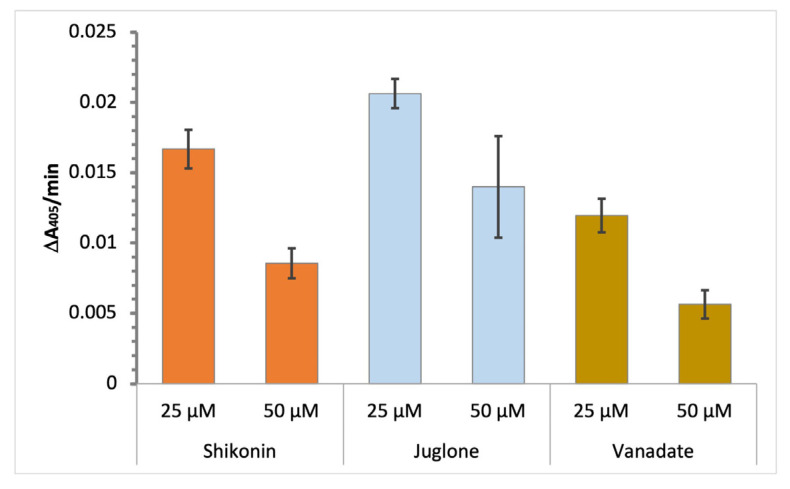
In vitro inhibition of Mt-PTPa via naphthoquinone derivatives shikonin and juglone. Comparison of the inhibition of Mt-PTPa via shikonin and juglone with that of the known competitive inhibitor of phosphatases, vanadate. Reactions were carried out as described in the Figure 1 legend. The hydrolysis of pNPP (100 µM) via Mt-PTPa (10 µM) in the presence of different concentrations of each compound (25 and 50 µM) was investigated in a total reaction volume of 100 µL. Values are shown as mean ± SD (*n* = 3).

Figure 3 shows that juglone has an inhibitory effect on Mt-PTPa comparable to that of shikonin. As a control, we included vanadate, a known competitive inhibitor of phosphatases, to give an indication of the potency of shikonin and juglone as inhibitors of Mt-PTPa. The results show that at 25 and 50 µM, the two compounds show comparable levels of inhibition to vanadate, a known active site inhibitor of enzymes that mediate phosphoryl transfer reactions [18,19].

### 3.4. Naphthoquinones Inhibit Mt-PTPa via a Mixed Inhibition Mechanism

**Figure 4:**
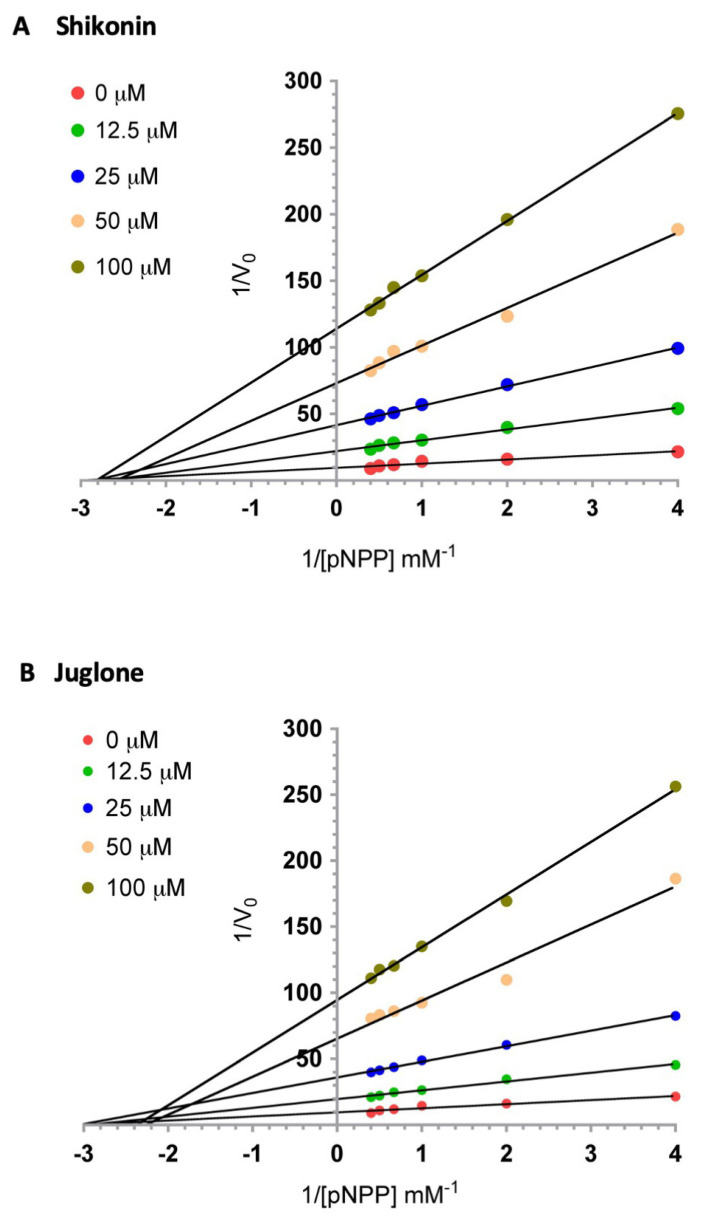
Double reciprocal plot analysis of the mechanism of Mt-PTPa inhibition via (**A**) shikonin, and (**B**) juglone. The effect of different concentrations of substrate pNPP (0.25, 0.5, 1.0, 1.5, 2.0, and 2.5 mM) on Mt-PTPa (10 µM) activity in the presence of different concentrations of each inhibitor compound (0, 12.5, 25, 50, and 100 µM) was investigated in a total reaction volume of 100 µL.

To gain more insights into how the two naphthoquinones inhibit Mt-PTPa, we investigated the effect of phosphatase substrate concentration (0.25–2.50 mM pNPP) on the pattern of inhibition by shikonin and juglone. The results are compatible with a non-competitive inhibition pattern for both compounds, since increasing substrate concentration did not relieve the inhibition based on the double-reciprocal analysis of the data (Figure 4). A Dixon plot analysis [20] of the data shown in Figure 4 gave inhibitor constants (Ki) of 8.5 µM and 12.5 µM for shikonin and juglone, respectively (Table 1). These values are comparable to what has been reported for PTP1B [21], a mammalian PTP that is considered a therapeutic target for cancer and other conditions [22,23,24].

## 4. Discussion

The potential development of antitubercular drugs based on *Mtb* LMW-PTPs (Mt-PTPa and Mt-PTPb) has consistently remained a key focus of active research [7,13,25,26,27,28], and more recently, there has been renewed interest in PTPs in general as therapeutic targets due to their key roles in biochemical processes and their regulations [29,30,31,32].

We started by screening the Screen-Well^®^ Natural Product library in search of compounds that inhibit Mt-PTPa at micromolar concentrations. The initial screen identified epigallocatechin, myricetin, rosmarinic acid, and shikonin as hits based on the set criteria. Further concentration-dependent investigation identified shikonin as the main promising inhibitor among the four compounds (Figure 1), while the IC_50_ values obtained for epigallocatechin, myricetin, and rosmarinic acid were not low enough to justify further investigation. Hence, we did not carry out further characterisation of these compounds in the present work. The strong inhibitory effect shown by shikonin and its reported inhibition of PTPs prompted us to investigate the effect of another structurally related naphthoquinone, juglone (5-hydroxy-1,4-naphthoquinone). Further concentration-dependent analysis confirmed that juglone also strongly inhibited Mt-PTPa (Figure 4; Table 1). Double-reciprocal and Dixon plots confirmed that the two naphthoquinones were non-competitive or mixed inhibitors of Mt-PTPa.

Shikonin is a bioactive compound from the roots of the Zicao plant (*Lithospermum erythrorhizon*) and has gained interest due to its extensive pharmacological effects [33]. It has been used in traditional Chinese medicine to treat infections, inflammation, and haemorrhagic diseases for >2000 years [34]. Studies have also reported its potential anticancer effects, including the inhibition of cell proliferation, the induction of apoptosis, and the suppression of cell migration [33,35]. Other studies report that shikonin exhibits diverse pharmacological properties, such as anti-inflammatory and antitumor effects [36,37,38,39].

The inhibition of CDC25 phosphatases via naphthoquinones has been implicated in several studies [40,41,42]. Saeed et al. [21] demonstrated that shikonin displayed powerful inhibitory effects against protein tyrosine phosphatase 1B (PTP1B) in vitro, with an IC_50_ value of 15.51 μM. In comparison, ursolic acid, a known strong inhibitor of PTP1B, gave an IC_50_ of 8.72 μM. Kinetic analysis carried out in the study led to the conclusion that shikonin inhibited PTP1B via a competitive inhibition mechanism. Zhang et al. [43] showed that shikonin and its analogues inhibited recombinant human Cdc25A, -B, and -C phosphatases in vitro in a concentration-dependent manner, with IC_50_ values ranging from 2.14 to 14.32 μM. Molecular docking carried out in the study suggested that shikonin bound to an inhibitor (allosteric) site away from the active site. Similarly, Kabacki et al. [44] showed that napthoquinones inhibited CDC25 phosphatase via a mixed-inhibition-type mechanism via the reversible oxidation of cysteine residues. Double-reciprocal plot analysis of our data gave a pattern consistent with the mixed inhibition mechanism, in agreement with conclusions reached by Kabacki et al. [44] and Zhang et al. [43].

While there are no reports on the inhibition of PTPs via juglone specifically, several studies have linked the cellular effects of naphthoquinones to the inhibition or inactivation of protein tyrosine phosphatases. Abdelmohsen et al. [45] and Beier et al. [46] found that the naphthoquinone compound, menadione, inhibited PTPs by dephosphorylating EGFR and ErbB2. In another study, Östman et al. [47] showed that menadione activates ErbB receptor tyrosine kinases, leading to PTP inactivation. In neutrophils, a shikonin derivative, acetylshikonin, reduced N-formyl-L-methionyl-L-leucyl-L-phenylalanine-induced protein tyrosine phosphorylation by 90% [48]. On the other hand, in a screen of small molecules with insulin-like actions, Kamei et al. [49] demonstrated that shikonin effectively enhances glucose uptake in 3T3L1 adipocytes and rat cardiomyocytes, with minimal impact on protein tyrosine phosphorylation within these cells.

The findings reported in this study show that naphthoquinones could be a group of compounds with specific allosteric inhibitory effects on the *Mycobacterium tuberculosis* protein tyrosine phosphatase A. Ribiero et al. [50] reviewed the potential for developing CDC25 inhibition via the naphthoquinone menadione for therapeutic applications. Yoshikawa et al. [51] showed that menadione inhibits another PTP, PTEN, both in vitro and in vivo. Cao et al. [52] reported the inhibitory effect of naphthoquinones against a wider range of PTPs, including MKP-1 and MKP-3, and Perron et al. [53] reported an allosteric non-competitive mechanism for a naphthoquinone derivative that inhibited CD45 tyrosine phosphatase.

Prokaryotic PTPs can be categorised into three families: (1) eukaryotic-like phosphatases, or dual-specificity PTPs (DSPs); (2) polymerase-histidinol phosphatases (PHPs); and (3) low-molecular-weight PTPs (LMW-PTPs) such as Mt-PTPa [54], with bacterial DSPs and LMW-PTPs also sharing the C(X)_5_R motif within the catalytic domain. Studies of the catalytic mechanism of Mt-PTPa show that ligand binding induces a conformational rearrangement of the D-loop around the active site [55]. Niesteruk et al. [56] showed that the oxidative inactivation of the catalytic cysteine residue is linked to conformational changes at the active site, leading to the temporary inactivation of the enzyme. As mentioned earlier, the initial cleavage step of the phosphoryl transfer reaction is mediated by the active site cysteine 11 (C11) located in the well-conserved P-loop [(H/V)CX5R(S/T)] characteristic of PTPs [57]. This catalytic cysteine in the P-loop is proposed to act as a redox switch that regulates the phosphatase activity of Mt-PTPa [56]. This could explain the mixed inhibition pattern by the two naphthoquinones observed in this study. Shikonin and juglone have been linked with cysteine-targeted protein inactivation. Several studies reporting the inhibition of enzyme activity via juglone associate the effect with the protein inactivation mechanism, usually via the modification of cysteine side chains at residues outside the active site [58,59,60,61,62].

A combination of experimental analysis and computational modelling shows that Mt-PTPa is subject to allosteric control, with some mutations predicted to have potential activation effects [63]. Similar susceptibility to allosteric modulation was reported by Stefan et al. [64], in which Mt-PTPa was shown to undergo a conformational change that leads to allosteric substrate activation.

It is noteworthy that while we have found two naphthoquinones as inhibitors of Mt-PTPa in this study, earlier studies have identified other compounds that have specific inhibitory effects on Mt-PTPa. Examples include synthetic chalcones [65,66], phenyl azole derivatives [67], thiosemicarbazides [12], and Cis-2 and trans-2-eicosenoic fatty acids [68].

It would be informative to identify the specific sites on Mt-PTPa that shikonin and juglone interact with to mediate their non-competitive inhibition of phosphatase activity. Further studies are needed to understand the molecular mechanism of naphthoquinone’s inhibition of Mt-PTPa and characterise the specific structural features of the compounds that interact with the enzyme. A detailed characterisation of the effects of a wider range of naphthoquinones would be required to fully establish the case for developing them as potential therapeutics for managing tuberculosis.

## Figures and Tables

**Table 1:** IC_50_ values and inhibition constants of Mt-PTPa inhibitors used in this study.

	IC_50_ (µM)	K_I_ (µM)
Rosmarinic Acid	900 ± 132	ND
Epigallocatechin	492 ± 101	ND
Myricetin	250 ± 25	ND
Shikonin	33 ± 7	8.5
Juglone	ND	12.5

The details for the determination of the different constants are described in the Results section. Values are shown as mean ± SD (*n* = 3).

## Data Availability

The data used to support the findings of this study are provided within this article. Further information can be provided by the corresponding author upon request.
